# Efficacy and safety of two different adjuvant chemotherapy regimens in combination with concurrent chemoradiotherapy in treating patients with advanced nasopharyngeal carcinoma: A protocol for randomized controlled trial

**DOI:** 10.1097/MD.0000000000025980

**Published:** 2021-05-28

**Authors:** Lu-Lu Zhang, Jun Cui, Hong-Fei Tan, Zheng Xiao, Li-Xia Pan

**Affiliations:** a Department of Nephrology; b Department of Respiratory; c Department of Sports Medicine; d Department of Emergency; e Department of Pathology, Wuhan Fourth Hospital; Puai Hospital, Tongji Medical College, Huazhong University of Science and Technology, Wuhan, Hubei Province, China.

**Keywords:** chemoradiotherapy, cisplatin, 5-fluorouracil, gemcitabine, nasopharyngeal carcinoma

## Abstract

**Background:**

Concurrent chemoradiotherapy is widely utilised as a standardized primary method of treatment for patients with advanced nasopharyngeal carcinoma (NPC). However, the combination of concurrent chemoradiotherapy and adjuvant chemotherapy for treating NPC patients remain unclear. Therefore, this study attempts to elucidate the efficiency and safety of concurrent chemoradiotherapy combined with adjuvant chemotherapy (gemcitabine plus cisplatin versus 5-fluorouracil plus cisplatin) for treating patients with NPC.

**Materials and Methods:**

This study is a randomized, multicentral, open-labelled trial to assess the clinical efficiency and safety of using concurrent chemoradiotherapy combined with adjuvant chemotherapy as a therapeutic measure for advanced NPC patients. A total of 50 patients will be randomly assigned into 2 groups, namely treatment-group-one and treatment-group-two. Eligible patients will be administered with concurrent chemoradiotherapy and subsequentially with adjuvant chemotherapy (gemcitabine plus cisplatin or 5-fluorouracil plus cisplatin). Moreover, the primary endpoint is a comparison of progression-free survival between concurrent chemoradiotherapy and subsequentially adjuvant gemcitabine and cisplatin and chemoradiotherapy, which is proceeded by adjuvant 5-fluorouracil and cisplatin in advanced NPC patients. Overall survival, overall response rate, incidence of acute and late toxicity, and adverse events are the minor endpoints. Statistical analyses will be performed with SPSS 25.0 software.

**Discussion:**

The current research evaluates the clinical efficiency and safety of utilising concurrent chemoradiotherapy combined with adjuvant chemotherapy as a therapeutic strategy to treat advanced NPC patients. The work done in this study will provide a clinical basis for concurrent chemoradiotherapy in combination with adjuvant chemotherapy for treating advanced NPC.

**Trial registration:**

DOI 10.17605/OSF.IO/5UPVM.

## 1 Introduction

Nasopharyngeal carcinoma (NPC) is a malicious form of tumor that emerges from the nasopharyngeal epithelial lining of the nasopharynx with a distinct geographical spread and racial prevalence. GLOBOCAN cancer estimated nearly 130,000 fresh NPC cases in 2018, which is nearly 0.7% of worldwide cancer cases in 2018, with the highest incidence rates in regions in North Africa, Southeastern Asia, and South China.^[1]^ Numerous advancements in treatment methods, such as immunotherapy, chemotherapy, and radiotherapy have been widely used over the last 10 years to treat NPC patients. However, the clinical prognosis for NPC patients is still low, nearly 20% of NPC cases go on to face recurrences and the overall survival rate after 5 years is between 50% to 70%.^[2]^ Thus, coming up with novel combinations of chemotherapies to improve the prognosis of advanced NPC patients within tolerable toxicity is urgently needed.

Recently, numerous randomized clinical trials, have provided confirmation of the significance behind combining different forms of chemotherapy to treat advanced NPC.^[3–6]^ Admittedly, treating advanced NPC cases with cisplatin-based concurrent chemoradiotherapy has had a considerable effect on prolonging the survival of advanced NPC patients. Cisplatin is related to lack of treatment compliance and standard of life due to its known side-effects. Furthermore, cisplatin-based concurrent chemoradiotherapy necessitates pre- and posttreatment hydration for renal protection throughout the cisplatin administration, which tends to increase hospitalization periods. The introduction of Intensity-Modulated Radiotherapy (IMRT) has substantially improved local control and distant metastasis is currently the primary reason behind treatment failure.^[7–9]^ Additional developments in systemic control via concurrent chemotherapy seems far-off, mainly due to the toxic effects. Therefore, it is crucial to consider adjuvant chemotherapy to advance therapy.^[10]^ To this end, the present study attempts to comparatively study the efficiency and safety of utilising concurrent chemoradiotherapy proceeded by adjuvant chemotherapy (gemcitabine plus cisplatin vs 5-fluorouracil plus cisplatin) as a therapeutic strategy for NPC cases.

## 2 Materials and methods

### 2.1 Study design and setting

**Figure 1:**
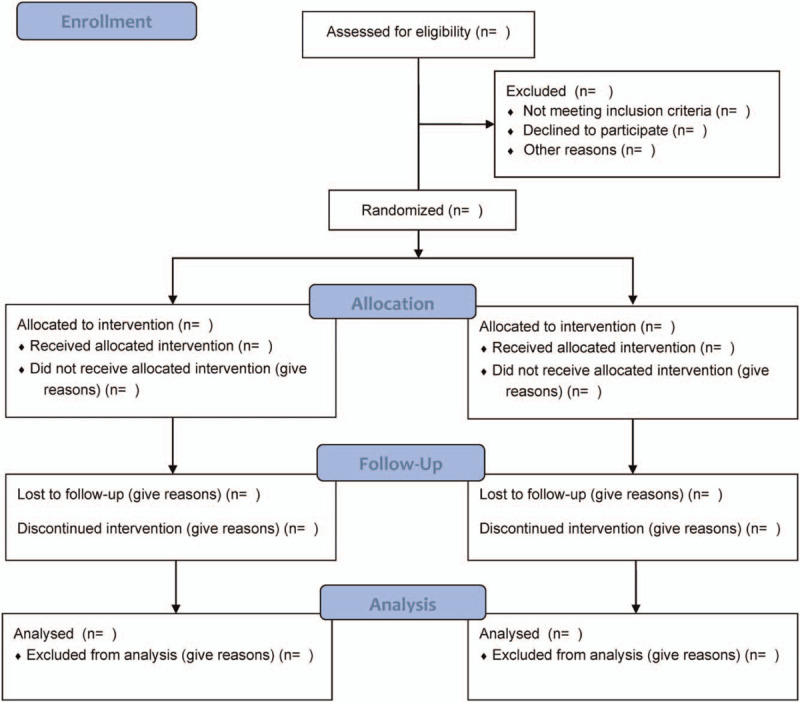
Flow diagram of the study.

The present randomized control trial will be a multicentral and open-labelled aimed at evaluating the clinical efficiency and safety of adopting concurrent chemoradiotherapy proceeded by adjuvant chemotherapy (gemcitabine plus cisplatin vs 5-fluorouracil plus cisplatin) as a therapeutic strategy for cases with histologically diagnosed advanced NPC. This study will involve 50 participants (patients) from Wuhan Fourth Hospital; Puai Hospital, Tongji Medical College, Huazhong University of Science and Technology, Wuhan, Hubei Province, China. Eligible patients will be randomized under a 1:1 ration into 2 groups, namely treatment group 1 and treatment group 2, which will receive concurrent chemoradiotherapy in combination with adjuvant chemotherapy (gemcitabine plus cisplatin or 5-fluorouracil plus cisplatin). The present study will be conducted according to the Standard Protocol Recommendations for Interventional Trials 2013 Statement.^[11]^ The flow chart is shown in Figure 1.

### 2.2 Ethics and registration

The current study adheres to the Helsinki Declaration and it was approved by the Ethics Committee of Wuhan Fourth Hospital; Puai Hospital, Tongji Medical College, Huazhong University of Science and Technology. The present study protocol is registered on the Open Science Framework (OSF, registration number: 10.17605/OSF.IO/5UPVM). Each participant is required to sign a written informed consent form.

### 2.3 Participants

All patients with histologically diagnosed advanced NPC will be recruited. The following criteria of inclusion and exclusion are utilized:

#### 2.3.1 Inclusion criteria

a) Participants with fresh histologically established non-keratinizing (based on the World Health Organization histological form);
b) Tumor staged as T3-4N1/N2-3/M0-1 (in accordance with the American Joint Committee on Cancer 7th edition);
c) Satisfactory performance status: Karnofsky scale ≥70;
d) Nominal liver function assessment: Alanine Aminotransferase, Aspartate Aminotransferase less than 1.5 times upper limit of normal (ULN) concomitant with alkaline phosphatase less than 2.5 times ULN, and bilirubin less than ULN;
e) Sufficient renal functionality: creatinine clearance more than 60 ml/min;
f) Adequate marrow: haemoglobin more than 90 g/L, leucocyte count more than 4.0 × 10^3^ /μL, and platelet count more than 100.0 × 10^3^ /μL;
g) Willingness to sign a written informed consent form.

#### 2.3.2 Exclusion criteria

a) Participants with clinically significant, uncontrolled heart disease;
b) Treatment with palliative intent;
c) Pregnant participants or lactating patients;
d) A history of radiotherapy (apart from non-melanomatous types of skin cancer separate from projected radiotherapy treatment volume);
e) A history of chemotherapy or operation to prime tumor or lymph nodes (except diagnostic);
f) Known HIV infection or history of HIV infection independent from the cellular immune status;
g) Unwillingness to sign a written informed consent form.

### 2.4 Interventions

Patients divided into treatment group 1 will receive concurrent chemoradiotherapy (concurrent cisplatin 100 mg/m^2^ per 21 days for 3 cycles throughout IMRT), proceeded with adjuvant gemcitabine (1000 mg/m^2^ on the first day and eighth day) and cisplatin (80 mg/m^2^ on the first day) per 21 days for 3 cycles 4 weeks following radiotherapy. Patients in treatment group 2 will receive concurrent chemoradiotherapy (concurrent cisplatin 100 mg/m^2^ per 21 days for 3 cycles during IMRT), proceeded by adjuvant 5-fluorouracil (1000 mg/m^2^ continuous intravenous infusion 96 hours) and cisplatin (80 mg/m^2^ on the first day) per 28 days for 3 cycles 4 weeks following radiotherapy.

### 2.5 Outcomes

The major endpoint of the present study is a comparison of progression-free survival between concurrent chemoradiotherapy proceeded by adjuvant gemcitabine and cisplatin and chemoradiotherapy proceeded by adjuvant 5-fluorouracil and cisplatin in advanced NPC cases (survival that is independent from progressive disease is determined from the randomization date to the first day of progress at a particular site or fatality from any reason or censored at the date of the last follow-up). Meanwhile, the secondary endpoints are overall survival, overall response rate, incidence of acute and prolonged toxicity, and adverse events.

### 2.6 Randomization and blinding

The participants will be arbitrarily assigned to treatment group 1 and treatment group 2 in a ratio of 1:1 according to a computer-generated, blocked, random-allocation sequence. The present study protocol is an open-labelled trial. Thus, clinicians, patients, and data collectors cannot be blinded throughout the study. However, an autonomous result assessor will be blinded during the study to keep study bias to a minimum.

### 2.7 Statistical analysis

Each participant will be arbitrarily assigned into a group that will be involved in the central efficiency evaluation. The safe populace was defined as all participants who were administered with concurrent chemoradiotherapy combined with a minimum of 1 adjuvant chemotherapy cycle (gemcitabine plus cisplatin versus 5-fluorouracil plus cisplatin). This study will entail the safe populace in the safety analysis. The log-rank test will be utilized to calculate the key difference in the progression-free survival and overall survival between 2 groups. The numerical data of normal distribution will be evaluated via a nonpaired test. The comparison of categorical variables will be completed by a Pearson Chi-Squared test. All statistical testing was two-sided, and *P* < .05 will be regarded as statistically significant. The SPSS software (version 24.0) will be used to perform statistical analysis.

## 3 Discussion

Nasopharyngeal Carcinoma (NPC) is unlike other forms of head and neck cancers. NPC is different in its relation with the Epstein-Barr virus, malicious normal behavior and elevated risk of distant metastases.^[12,13]^ In excess of 95% of NPC cases have poor carcinoma and nearly 70% existent with locoregionally progressed condition in endemic areas.^[14,15]^ Adjuvant cisplatin and 5-fluorouracil chemotherapy is the suggested treatment strategy for advanced NPC patients.^[12]^ However, it is likely that the combination is only conducive for patients suffering from lower distant tumor. The widespread adoption of IMRT has substantially enhanced local control and distant metastasis remains the primary reason of negative treatment outcomes. NPC treatment should aim to enhance survival and diminish the toxicity due to treatment.

As mentioned above, a multicentral, randomized, open-labelled trial will verify the clinical efficiency and safety of concurrent chemoradiotherapy in combination with adjuvant chemotherapy (gemcitabine plus cisplatin or 5-fluorouracil plus cisplatin) for treating advanced NPC patients. Meanwhile, it is also hoped that the present study will open a new direction for treating advanced NPC.

## Author contributions

**Conceptualization:** Lu-Lu Zhang.

**Data curation:** Li-Xia Pan.

**Formal analysis:** Lu-Lu Zhang, Zheng Xiao, Li-Xia Pan.

**Investigation:** Jun Cui, Zheng Xiao.

**Methodology:** Jun Cui, Li-Xia Pan.

**Project administration:** Jun Cui, Hong-Fei Tan.

**Software:** Lu-Lu Zhang, Jun Cui, Zheng Xiao.

**Supervision:** Hong-Fei Tan.

**Validation:** Lu-Lu Zhang.

**Visualization:** Hong-Fei Tan.

**Writing – original draft:** Lu-Lu Zhang, Li-Xia Pan.

**Writing – review & editing:** Li-Xia Pan.

## References

[1] Bray F Ferlay J Soerjomataram I Siegel RL Torre LA Jemal A. Global cancer statistics 2018: GLOBOCAN estimates of incidence and mortality worldwide for 36 cancers in 185 countries. CA Cancer J Clin 2018;68:394–424. 30207593 10.3322/caac.21492

[2] Bensouda Y Kaikani W Ahbeddou N Rahhali R Jabri M Mrabti H. Treatment for metastatic nasopharyngeal carcinoma. Eur Ann Otorhinolaryngol Head Neck Dis 2011;128:79–85. 21177151 10.1016/j.anorl.2010.10.003

[3] Chen L Hu CS Chen XZ Hu GQ Cheng ZB Sun Y. Concurrent chemoradiotherapy plus adjuvant chemotherapy versus concurrent chemoradiotherapy alone in patients with locoregionally advanced nasopharyngeal carcinoma: a phase 3 multicentre randomised controlled trial. Lancet Oncol 2012;13:163–71. 22154591 10.1016/S1470-2045(11)70320-5

[4] Sun Y Li WF Chen NY Zhang N Hu GQ Xie FY. Induction chemotherapy plus concurrent chemoradiotherapy versus concurrent chemoradiotherapy alone in locoregionally advanced nasopharyngeal carcinoma: a phase 3, multicentre, randomised controlled trial. Lancet Oncol 2016;17:1509–20. 27686945 10.1016/S1470-2045(16)30410-7

[5] Tang LQ Chen DP Guo L Mo HY Huang Y Guo SS. Concurrent chemoradiotherapy with nedaplatin versus cisplatin in stage II-IVB nasopharyngeal carcinoma: an open-label, non-inferiority, randomised phase 3 trial. Lancet Oncol 2018;19:461–73. 29501366 10.1016/S1470-2045(18)30104-9

[6] Zhang Y Chen L Hu GQ. Gemcitabine and cisplatin induction chemotherapy in nasopharyngeal carcinoma. N Engl J Med 2019;381:1124–35. 31150573 10.1056/NEJMoa1905287

[7] Zhang MX Li J Shen GP. Intensity-modulated radiotherapy prolongs the survival of patients with nasopharyngeal carcinoma compared with conventional two-dimensional radiotherapy: A 10-year experience with a large cohort and long follow-up. Eur J Cancer 2015;51:2587–95. 26318726 10.1016/j.ejca.2015.08.006

[8] Co J Mejia MB Dizon JM. Evidence on effectiveness of intensity-modulated radiotherapy versus 2-dimensional radiotherapy in the treatment of nasopharyngeal carcinoma: meta-analysis and a systematic review of the literature. Head Neck 2016;38: Suppl 1: E2130–42. 25546181 10.1002/hed.23977

[9] Lai SZ Li WF Chen L. How does intensity-modulated radiotherapy versus conventional two-dimensional radiotherapy influence the treatment results in nasopharyngeal carcinoma patients? Int J Radiat Oncol Biol Phys 2011;80:661–8. 20643517 10.1016/j.ijrobp.2010.03.024

[10] Lee AW Tung SY Chua DT. Randomized trial of radiotherapy plus concurrent-adjuvant chemotherapy vs radiotherapy alone for regionally advanced nasopharyngeal carcinoma. J Natl Cancer Inst 2010;102:1188–98. 20634482 10.1093/jnci/djq258

[11] Chan AW Tetzlaff JM Gøtzsche PC Altman DG Mann H Berlin JA. SPIRIT 2013 explanation and elaboration: guidance for protocols of clinical trials. BMJ 2013;346:e7586. 23303884 10.1136/bmj.e7586 PMC3541470

[12] Chen YP Chan ATC Le QT Blanchard P Sun Y Ma J. Nasopharyngeal carcinoma. Lancet 2019;394:64–80. 31178151 10.1016/S0140-6736(19)30956-0

[13] Lee AW Lin JC Ng WT. Current management of nasopharyngeal cancer. Semin Radiat Oncol 2012;22:233–44. 22687948 10.1016/j.semradonc.2012.03.008

[14] Wang HY Chang YL To KF. A new prognostic histopathologic classification of nasopharyngeal carcinoma. Chin J Cancer 2016;35:41. 27146632 10.1186/s40880-016-0103-5 PMC4857443

[15] Mao YP Xie FY Liu LZ. Re-evaluation of 6th edition of AJCC staging system for nasopharyngeal carcinoma and proposed improvement based on magnetic resonance imaging. Int J Radiat Oncol Biol Phys 2009;73:1326–34. 19153016 10.1016/j.ijrobp.2008.07.062

